# Breast cancer relatives’ physical activity intervention needs and preferences: qualitative results

**DOI:** 10.1186/s12905-017-0392-0

**Published:** 2017-05-19

**Authors:** Sheri J. Hartman, Rochelle K. Rosen

**Affiliations:** 1 Department of Family Medicine and Public Health, University of California, San Diego, USA; 2 Cancer Prevention and Control Program, Moores UCSD Cancer Center, University of California, San Diego, USA; 3 Centers for Behavioral and Preventive Medicine, The Miriam Hospital, Providence, USA; 4 Department of Behavioral and Social Science, Brown School of Public Health, Providence, USA

**Keywords:** Qualitative methods, Breast cancer risk, Physical activity, Intervention development

## Abstract

**Background:**

While many risk factors for breast cancer, such as family history, are not modifiable, some, however, can be modified. The study used formative qualitative research to learn about the physical activity intervention preferences and needs of first-degree female relatives (FDFRs) of breast cancer patients; that information was then used to develop a targeted physical activity intervention.

**Methods:**

Twenty FDFRs first completed a 12-week physical activity intervention and then attended two sequential focus groups (7 groups total). In the first set of focus groups participants provided feedback on the intervention. In the follow-up focus groups, proposed changes based on collected responses from the first groups were presented and participants provided feedback to further refine the intervention.

**Results:**

Overall, we found strong interest for an intervention using breast cancer-related health concerns to promote positive behavior change. A theme underlying all of the feedback was the desire for a personalized intervention that was directly relevant to their lives. Participants wanted this personalization achieved through individually tailored content and incorporation of stories from other FDFRs. In order to successfully use concerns about breast cancer to motivate behavior change, participants also wanted a discussion about their individual risk factors for breast cancer including, but not limited to, lack of physical activity.

**Conclusions:**

This study demonstrates women’s interest in receiving personalized information and highlights specific ways to individualize an intervention that increases motivation and engagement. Using a sequential qualitative approach was effective for formative intervention development.

**Trial registration number:**

NCT03115658 (Retrospectively registered 4/13/17).

## Background

Breast cancer is the second leading cause of cancer death among women, and women with a family history are at increased risk for developing breast cancer [1]. According to two meta-analyses [2, 3], women with one first degree female relative (FDFR) with breast cancer have approximately double the risk of developing breast cancer compared to women with no family history of the disease. Although women cannot modify some of their risk factors for breast cancer, including family history and age, other risk factors, including physical activity, can be changed. Identifying preferences and needs regarding a physical activity intervention in this high-risk subgroup of women is important for developing targeted interventions to increase physical activity.

Data from numerous studies has consistently found an inverse relationship between participation in regular physical activity and breast cancer risk with, on average, a 20% risk reduction [4–9]. Research also supports a dose-response relationship between physical activity and breast cancer risk [6, 10]. One review [6] calculated that for every one-hour of physical activity per week there is an additional 6% reduction of lifetime breast cancer risk. In addition to the direct benefits of physical activity on breast cancer, physical activity can also reduce adiposity [11–13], which is associated with increased risk of lifetime breast cancer in postmenopausal women [14–17]. The American Cancer Society established physical activity recommendations for cancer prevention of engaging in at least 150 min of moderate intensity exercise or 75 min of vigorous intensity activity each week, or an equivalent combination, with the acknowledgement that higher amounts may provide even greater protection [18]. Therefore, promoting regular physical activity may be extremely important for women at increased risk for breast cancer.

While physical activity interventions have been developed for a variety of populations including healthy adults and cancer survivors, no interventions have been developed with a focus on first-degree relatives of cancer patients. While these individuals may be healthy themselves, they may also have different health concerns and motivations for exercising. Research suggests that changes in a health behavior are more likely when the intervention is tailored to motivators that are personally relevant to the target group [19–21]. FDFRs of breast cancer patients may benefit from a physical activity intervention that addresses specific concerns related to their increased risk for breast cancer and possible increased distress surrounding their cancer risk or family member’s cancer diagnosis. Having an intervention specifically targeted to FDFRs of breast cancer patients may be more appealing to these women than a standard intervention and therefore could be more effective at encouraging regular physical activity and provide motivation to maintain regular physical activity. Therefore, the first aim of the study was to use formative research to learn about the physical activity intervention preferences and needs of FDFRs of breast cancer patients. The second aim was to develop a targeted physical activity intervention based on the first round of focus groups and then present the intervention to the participants in a second round of focus groups to gain additional feedback for further intervention refinements.

## Methods

### Participants

Women with at least one FDFR (i.e., mother, sister, or daughter) diagnosed with breast cancer were recruited for this study. Eligible women had to be engaging in less than 90 min per week of moderate intensity physical activity as the intent was to develop an intervention for underactive FDFRs. Exclusion criteria included: personal diagnosis of any type of cancer except basal cell carcinoma; currently pregnant or intention to get pregnant in the next 3 months; presence of a known medical condition that would make it difficult/dangerous to exercise (e.g., cardiovascular disease); severe psychiatric illness; plans to move from the area in the next 6 months; or unable to read and speak English. Web and radio advertisements and a local news story were used to recruit participants. Written informed consent was obtained, and the protocol was approved by the institutional review board of The Miriam Hospital, RI.

### Procedures

Participants first completed a 12-week physical activity intervention, which had previously been developed for healthy men and women, and then attended two sequential focus groups. In the first set of focus groups participants provided feedback on the 12-week physical activity intervention. In the second focus groups participants returned to provide feedback on proposed changes to the invention that were made based on collected responses from the first groups.

The intervention was a 12-week theory-based and individually-tailored physical activity intervention shown to be efficacious with sedentary men and women [22–25]. At the in-person baseline visit participants were informed of the study goal to engage in at least 150 min of moderate intensity exercise per week; they then set individualized exercise goals and created a personalized exercise plan to do on their own. Participants were instructed how to self-monitor their exercise, and received information on exercising safely. All other contacts occurred through the mail with participants receiving tailored and non-tailored physical activity information weekly for the first month, and bi-weekly for months 2 and 3. Tailored physical activity information was based on participants’ responses to questionnaires measuring constructs from Social Cognitive Theory and the Transtheoretical Model including self-efficacy for physical activity, stage of readiness to be physically activity, and decisional balance for physical activity. Non-tailored materials consisted of a variety of physical activity focused topics from exercising safely to tips to stay motivated. In addition to the previously-developed physical activity intervention materials, participants received one breast cancer information sheet per month developed specifically for the current study. These addressed: 1) modifiable and non-modifiable breast cancer risk factors; 2) how physical activity relates to, and may lower, breast cancer risk; 3) and breast cancer screening. Twenty-seven women enrolled in the intervention [26]. Full details on this intervention are provided elsewhere [27].

A total of 7 focus groups were conducted with the women after the end of the intervention period, four in the first round, and three in the second round. Twenty women (4–6 per group) attended the first round of focus groups, in which we sought to understand their experience in the physical activity intervention and response to the new materials. We also asked how the intervention could be modified to better address the needs of FDFRs like themselves. An open-ended qualitative data collection guide was used to provide consistency across groups. The questions were designed to elicit specific feedback about current intervention and proposed changes in the future. The focus group guide questions and prompts were used as facilitative tools, rather than a rigid script, allowing emergent ideas to be raised.

Women who attended the first round of focus groups were invited back to attend the second round. Eighteen of the 20 women attended the second round focus groups (4–8 per group). In these groups we sought feedback on proposed modifications to the intervention that were developed based on the information learned through the first round of focus groups. A slide presentation was used to present the proposed modifications in comparison to the original intervention, and to show mocked up examples of new intervention components.

Focus groups were audio recorded, and field notes were taken at each focus group. The focus group guides were developed by PhD-level staff experienced in physical activity interventions, breast cancer research, and qualitative methods (S.J.H. and R.K.R.). Due to practical limitations of participants being limited to those who were enrolled in the physical activity intervention it was decided to hold the focus groups as long as no fewer than 4 participants attended. The facilitator (S.J.H.) was known to the participants because she did exercise goal setting with them in the intervention portion of their study participation. In each focus group the facilitator addressed this familiarity, explaining that she had not developed the intervention but had used protocols from a previous study on which she sought participant feedback. Participants were candid in their feedback and we believe that participants understood that they were asking to partner with the facilitator to respond to and help modify the PA intervention in which they participated. Debriefing was done after each focus group with the facilitator (S.J.H.) and note-taker to allow for iterative modification of the agenda and to assess data saturation.

Audio recordings were transcribed and each transcription reviewed by the facilitator (S.J.H.) to ensure accuracy. Transcripts were reviewed by two independent coders (S.J.H. and R.K.R.) using a coding guide developed from the focus group agendas to reflect key content areas and themes that emerged from the discussions. Separate coding guides were used for the first and second round of focus groups. Any discrepancies between the two coders were discussed and resolved. The agreed upon codes were entered in NVivo 10 [28] to aid in the data analysis. A thematic content analysis [29] was performed on these data using a qualitative descriptive approach [30]. Our analytical goal was to understand both the most common responses to the intervention experience and any unique ones, with particular attention to whether and how being a FDFR shapes attitudes and experience with physical activity generally, and with the intervention specifically.

## Results

Participants (*n* = 20) were a mean age of 40 years old (SD = 11.01; range 21-54 years) predominantly identified as non-Hispanic White (85%) had college or graduate level education (60%) and had an annual income greater than $40,000 US dollars (66%). Seventeen reported that their mother had been diagnosed with breast cancer, two had sisters with breast cancer, and one woman had both a mother and sister diagnosed with breast cancer. Participants reported engaging in a mean of 30.25 min/week (SD = 33.07) of moderate to vigorous physical activity at baseline, that increased to an average of 180.25 min/week (SD = 137.80) of moderate to vigorous physical activity at the end of the 12 week intervention. Fifty-five percent of participants reported engaging in at least 150 min of moderate intensity exercise a week (See Table 1).

**Table 1:** Participant characteristics (*n*=20)

	Intervention Arm (*N* = 20) % or *Mean* (*SD*)
Age, years	40 (11.01)
Education	
Partial College or Less	40%
College graduate	40%
Graduate degree	20%
White	95%
Non-Hispanic or Latino	85%
Number of first-degree relatives with breast cancer	
1	95%
2	5%
Breast cancer patient’s relationship to the participant	
Mother	85%
Sister	10%
Mother & Sister	5%
Income*	
< $40,000 USD	33%
$40,000–$59,999 USD	22%
≥ $60,000 USD	44%
Moderate to vigorous physical activity (min/week)	
Baseline	30.25 (33.07)
12-weeks	180.25 (137.80)
*n = 18	
USD = United States Dollar	

Participants provided feedback on the intervention including what motivated or did not motivate them to increase physical activity. They were also asked for ways the intervention could be improved to make it more relevant to women with a family history of breast cancer. Five key themes developed from the coding and analysis. Two of these areas, *breast cancer risk as a motivator to exercise* and *timing of the intervention relative to the relative’s cancer diagnoses,* were topics from the focus group guide. The other three areas, *discussion of personalized breast cancer risk factors*, *adding personal stories as sources of information and motivation*, and *adding general breast cancer information* were topics that emerged from the discussions. In the discussion that follows, quotes from the focus groups are used to illustrate participant comments that were used to develop each theme.

1. **Breast cancer risk as motivator to exercise**
  Overall the women said knowing that physical activity was a way to reduce breast cancer risk provided them with a motivation to exercise. “*It’s just… like osteoporosis: if you know that weight bearing exercise keeps you from having your bones break, that actually may change your mindset. And if you really believe that exercising more will truly had an impact on breast cancer, then, you know, it’s that for me. I’ll do anything if I know the reason”* (53yo, sister BC). Many participants, however, told us that this information was not motivating in the study, because the only connection between physical activity and breast cancer was in information sheets mailed out to them; that is the information could have been better integrated into the existing intervention materials and discussions. One woman stated: “*We got these things but we never talked about them when we met with you either time so I think it didn’t connect at all because of that, because you didn’t talk about it*” (23yo, mother BC).
  To address this feedback we proposed in the second round of focus groups to change the baseline in-person meeting, which previously focused only on physical activity, to also include a discussion about the relationship of breast cancer risk to physical activity. The women were very supportive of this idea: *“I think it’ll bring it together. When I first did it I didn’t get the feeling that breast cancer or anything about cancer was a part of the program much, so I think that’ll bring it nice and up to the front”* (48yo, mother BC).
2. **Discussion of personalize breast cancer risk factors**
  Almost all participants were interested in having a discussion at the start of the study about their personal risk factors for breast cancer. They were interested in a discussion that covered not only physical activity, but other modifiable and non-modifiable breast cancer risk factors as well. For example one woman stated: “*After you get our information you could be like, ‘well you’re 26, your mother had breast cancer at this age. This is what you should think about doing in 5 years. This is what you should do now’*” (28yo, mother BC). They wanted a personalized discussion in order to understand their own level of breast cancer risk. One woman said: “*Just how worried really should I be? That was one of the questions on your form: ‘How much does this affect you?” And um, not a lot, but maybe it should”* (53yo, sister BC). In response, another woman said: “*It affects me a lot and maybe it shouldn’t”* (23yo, mother BC).
  To address this issue, we proposed incorporating a discussion about breast cancer risk factors tailored to participants’ individual risk factors including current weight, physical activity level, and menopausal status into the baseline session. Participants in the second set of focus groups were enthusiastic about this addition.
3. **Add personal stories from other FDFRs**
  Many of the women thought incorporating personal stories throughout the intervention would increase motivation. They wanted to hear from women like themselves, other women whose family member had breast cancer, about increasing physical activity. They also wanted to hear about those women’s experiences with their family member’s cancer diagnosis and treatment. The women felt that seeing women like themselves be successful in increasing their physical activity could be motivational to their own success. The women suggested this could be accomplished by adding personal stories to the materials. They indicated that such stories would help “*bring life to it [the print materials]”* (46yo, mother BC). They also said: “*paper takes the emotion of it*” (51yo, mother BC), and that print “*tends to sink in more when you can put a face or a name [to it]”* (47yo, mother BC). Another woman stated: “*It might be good to hear some other people’s stories like that. It might help someone just to hear the personal stories…it might even encourage us to actually read the stuff*” (48yo, mother BC). Another suggestion from the women was to also allow the participants to be able to share their personal stories with one another. For example, one woman said “*If there was like a website that people could put their personal stories on the website and you could go there and do them*” (48yo, mother BC).
  To address these issues, at the second round of focus groups we proposed incorporating personal stories throughout the materials. The participants were very supportive of this plan. We also proposed a message board where participants could share their personal stories with the other participants. The overall feedback about providing a message board was positive, but the response about actual anticipated use of it was mixed. About half were interested in using a message board and about half said they would not use one. However, the women who were not interested in posting on a message board still indicated that they would be interested in reading other people’s posts. For example, “*I would at least read it, and if I saw something on there that was interesting maybe I would respond*” (29yo, mother BC).
4. **Add general breast cancer information**
  Many of the women were also interested in information about breast cancer that was not related to physical activity. Some of the women reported having little information about breast cancer. *“I almost think that maybe more information about breast cancer to go with it would have been helpful. Because in my situation, my mother currently does have breast cancer…. I know basically that it puts me at a higher risk to get it, but that’s pretty much where my knowledge stops”* (28yo, mother BC). Another woman had outdated information: *“My mother died from breast cancer, so I had a lot of information. But it’s dated, because she died in 1996. So it would have been great for me to learn what they’ve learned since 1996”* (52yo, mother BC).
  One woman was not interested in general breast cancer information. She reporting having a high level of knowledge and suggested a personalized approach where information was provided based on an individual’s need: *“So when she was diagnosed I started collecting all sorts of information and actually my sister had it in 2001. So it was kind of like, I, oh for 10 years now it’s been circulating around my family. So for me I already knew more than I really wanted to know about breast cancer. And I kind of like, for me, it wasn’t going to be helpful for me personally. So perhaps an assessment of one’s like level of knowledge and understanding prior to enrollment in the study, might help to guide what materials are actually disseminated to people”* (38yo, mother and sister BC).
  Through the focus groups some of the women learned that the information they had was not accurate, which led to an interest in learning more about facts and myths about breast cancer and breast cancer risk. Several women also expressed an interest in receiving breast cancer related resources. This varied from books and suggested readings to local support groups for breast cancer survivors and their family members.
  Based on this feedback we proposed adding several types of breast cancer related information into the study and used the second set of focus groups to identify the specific topics they wanted covered in the new materials. Participants were interested in learning about genetic testing for breast cancer, information on free and low-cost mammograms, local breast cancer related talks (given by hospitals), questions to ask their doctor, breast cancer support groups and family member support groups, breast cancer related charity walks, mental health resources, and reputable breast cancer related websites.
5. **Timing of being in an exercise program relative to cancer diagnosis**
  Participants varied greatly regarding when their relative was diagnosed. This ranged from very recently diagnosed and currently in treatment to being diagnosed over 30 years ago. We therefore explored whether there was an optimal time to capitalize on concerns about breast cancer risk to motivate behavior change. Overall the feedback was mixed, with the general sense that it was a personal decision. *“So I think it’s whenever we’re ready to sign up for the program. If it’s being offered and we feel we’re strong enough to do it, go for it. And if you can provide us with information if we signed-up we’d know that this is what we are getting into, but I don’t think there would be a specific time because everybody’s situation’s different”* (23yo, mother BC). Some women felt there was no optimal period of time. For example: *“it’s been two years since my sister [was diagnosed], she just got her final full year clearance and I can’t think of a time when it would be more helpful or less helpful”* (53yo, sister BC). Several women stated that during a relative’s active treatment would not be optimal, since their priority at that time was helping their family. For example one woman said: *“It would have done no good while my mother was going through treatments because while it probably wasn’t the best, pretty much all of my energy was focused on her”* (32yo, mother BC). However another felt that being in a program could provide support for someone whose family member was just diagnosed. One woman expressed that it was not the time since diagnosed that mattered to her, but rather her FDFR’s age at diagnosis. She was currently the same age that her mother was when she was diagnosed with breast cancer. Although two other women whose mothers were diagnosed over 10 years ago felt some “*detachment*”, they thought it would have been more motivating had they enrolled closer to the time of diagnosis. Due to the wide range of opinions about when to enroll in an exercise program, we did not explore limiting enrollment by time since diagnosis, and did not discuss this topic further in focus group 2.

## Discussion

The current study sought to identify physical activity intervention preferences and needs of FDFRs. We found a strong interest for an intervention that used breast cancer related health concerns to promote positive behavior change. However, for this to be motivational the women wanted a personalized discussion at the beginning of the intervention to covered physical activity and other breast cancer risk factors. The women also wanted stories from other women like themselves to be incorporated into the materials to increase the interest and saliency of the information and to be able to share their own personal stories with other participants. In addition some of the women were interested in receiving general breast cancer information as they did not have current or accurate information; while those with up to date information were not interested in general breast cancer information. There was no consensus on the optimal time of when, in relation to their relatives breast cancer diagnosis, they would be most motivated to increase their physical activity; instead the women felt their readiness to change was a personal decision that would vary for each of them.

The concern underlying all of these findings is the desire for a personalized intervention that provides information on individual risk factors. The women wanted to feel that the intervention was directly relevant to them and their lives. In order to successfully use concerns about breast cancer to motivate increasing physical activity the women said they needed a personalized discussion about how physical activity could benefit them. They also wanted a discussion about other personally relevant breast cancer risk factors. In addition, the woman suggested greater overall personalization of the content of the intervention materials. This personalization took two forms: one, through the addition of stories from women like themselves or by contributing their own stories to the material, and two, by providing information on topics based on the women’s current knowledge base or interest. Not only did they feel that a more personalized approach would be more motivational, but that it would increase their engagement in the intervention itself.

This desire for personalization is not unique to FDFRs, but it highlights that tailoring an intervention to a concern that is personal to the participant, such as breast cancer risk was to these participants, may increase the participants’ motivation, increase the dose of the intervention they receive, and ultimately help motivate the desired behavior change. This is supported by other literature indicating that personalized interventions are more effective than standard interventions for producing behavior change [20, 31, 32]. Research also indicates that the more adherent a participant is to an intervention, the more successful they are at changing their behavior; [33, 34] therefore personalization may also enhance outcomes by increasing adherence.

Prior to the focus groups, participants were enrolled in a physical activity intervention, suggesting that they were at least somewhat interested in increasing their physical activity; therefore, the feedback may not generalize to a less motivated group. We purposely chose to obtain feedback from women who had experienced the intervention themselves, and who could provide specific feedback on what actually worked well and what could have been done more effectively. We felt this would give us more accurate and informative feedback than asking people to conceptualize what they would want in an intervention. Having the experience of a physical activity intervention, including receiving intervention materials and trying to change their own physical activity behavior allowed participants to provide rich and informative feedback on how to modify a physical activity intervention to better address the needs of our target population.

Although conducting sequential focus groups to gain in-depth feedback on developing a tailored physical activity intervention was a strength of this formative study, several limitations should be noted. The study had a small sample size which may limit the amount and range of feedback we received; however, it was appropriate for an exploratory study and during coding and analysis we found most topics to have rich data and felt that those data were saturated. The numbers attending some groups were relatively small, ranging from 4 to 6 overall. The facilitator was known to the participants; this was addressed at the outset of each group, to invite collaboration from the participants in the study aim of adapting an existing intervention. The sample consisted of mostly non-Hispanic White women, most were college educated, the majority of participants’ FDFR was their mother, and overall the sample was relatively young. These factors may limit the applicability of the results to more diverse populations. Future research should examine if intervention preferences found here are similar for sisters of breast cancer patients and for older women, whose concerns about breast cancer and risk for developing breast cancer may be more immediate than the current sample.

## Conclusions

This study supports personalization of materials to enhance motivation for behavior change. It also highlights specific areas and ways that personalization could be incorporated into interventions to increase motivation and engagement. While the details are specific to FDFRs the overall themes are likely generalizable to other groups. With the increase of technology being incorporated into interventions, future studies could be personalized to address the specific health concerns of that individual potentially using the feedback identified in the current study. Next steps are to test an intervention that incorporates personalization of content surrounding breast cancer and breast cancer risk to establish if these changes are effective at increasing adherence to physical activity guideline, provide physical and psychological benefits associated with being physically active and reduce cancer risk.

## Abbreviations

FDFRs: First-degree female relatives
Yo: years old.
BC: Breast cancer
